# Protective effects of melatonin and ghrelin on spermatogenesis: A narrative review of the literature

**Published:** 2017-05

**Authors:** Mohammadreza Gholami, Seyyed Amir Yasin Ahmadi, Abolfazl Abaszadeh, Arash Khaki

**Affiliations:** 1 *Department of Anatomy, Faculty of Medicine, Kermanshah University of Medical Sciences, Kermanshah, Iran.*; 2 *Student Research Committee, Lorestan University of Medical Sciences, Khorramabad, Iran.*; 3 *Department of Surgery, Lorestan University of Medical Sciences, Khorramabad, Iran.*; 4 *Department of Veterinary Pathology, Islamic Azad University, Tabriz Branch, Tabriz, Iran. *

**Keywords:** Spermatogonia, Cryopreservation, Vitrification, Melatonin, Ghrelin

## Abstract

Spermatocytogenesis starts from lumens of seminiferous cords and after migration to the basal membrane ends to the lumens again. We attempt to review the protective effects of melatonin and ghrelin on Spermatocytogenesis and in particular on spermatogonial stem cells, as two rather newly-discovered hormones. Testicular freezing prior to chemotherapy and radiotherapy is one of the ways of preserving fertility in children with cancer. The freezing has two methods of slow-freezing (cryopreservation) and rapid-freezing (vitrification). Administration of melatonin can maintain the quality of the germ cells underwent such processes, as well as ghrelin, can protect germ cells from the toxicities secondary to ischemic injuries, and pathologic apoptosis. This review indicates that in vitro or in vivo administration of melatonin or ghrelin, could be effective to preserve fertilization and also they can be used in assisted reproductive technologies to improve the quality of sperms. Future original studies should be propelled toward human studies, of course with observing the ethics.

## Introduction

Primordial germ cells (PGCs) are known as the origin of germ cells in both genders (1). However, most of the related studies have been done on mice. The origin of PGCs is still controversial, but it seems that they originate from an epiblastic tissue out of gonad and then migrate toward primordial genital ridge (2, 3). Development of PGCs is controlled by bone-morphometric proteins like the bone morphogenic protein-4 secreted by extra-embryonic ectoderm (4-6). In males, upon PGCs get to genital crest, they are surrounded by Sertoli cells and seminiferous cords are formed (7-9). At this time, the PGCs influenced by Notch signaling pathway of the Sertoli cells, are called as gonocyte (8, 10). 

**Figure 1:**
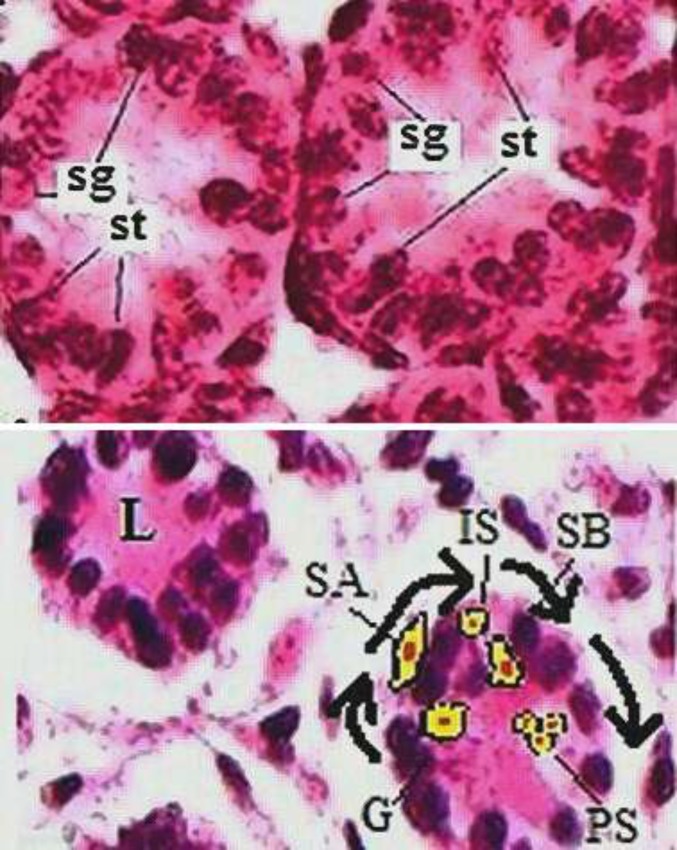
Spermatocytogenesis from the lumen to the lumen. L (lumen), PS (primary spermatocyte), G (gonocyte), SA (spermatogonia A), SB (spermatogonia B), IS (intermediate spermatogonia), sg (spermatogonia) and st (Sertoli cells) . The arrowed cells in yellow color, have been added graphically and schematically. Magnification ×400

The gonocytes keep on proliferation till arresting phase after the embryonic day 14.5 (in mice) (10). After birthday, the gonocytes move from lumen of the cords to the basal membrane (11). During the motion, the proliferation starts again and spermatogonium-A (SA) is formed. After that, the SAs get away from the basal membrane to the lumen again and during the motion, they create respectively intermediate spermatogonium and spermatogonium-B (SB) (12). Briefly, spermatocytogenesis stars from lumens of the cords and after migration to the basal membrane ends to the lumens again (Figure 1). 

SA formation, in turn, includes three levels; the A single spermatogonium (A_s_) which also is called as spermatogonial stem cell (SSC); and the next levels are respectively A paired spermatogonium (A_p_) and A aligned spermatogonium (A_al_) (12). Spermatogonial stem cells (SSCs) maintain their population through self-renewal (10, 13). The self-renewal can maintain the balance existing between A_s_ and A_p_. The ability to self-renewal is due to the fact that SSCs are undifferentiated like other stem cells (14, 15). The gene *inhibitor of DNA binding 4 *(*ID4*) is the involving gene in the mentioned balance. Reduction in expression of this gene is along with the reduction of SSC proliferation and infertility. The protein NANOS2 existing in A_s_ and A_p_, is also involved that its elimination or over-expression cause elimination or aggregation of SSCs (16). 

The inducing agent of SSC proliferation is glial cell line-derived neurotrophic factor (GDNF) and its signaling pathway released from Sertoli cells. Over-expression of this cascade results in aggregation of undifferentiated spermatogonia. Gradual elimination of SSCs could be secondary to either elimination of the GDNF or its receptors (17). Formation of intermediate spermatogonia and SBs is due to the protein spermatogenesis and oogenesis specific helix-loop-helix (SOHLH) (18). 

Spermatogenesis injuries could be due to the toxicity of chemotherapeutic drugs such as busulfan and cispelatin or toxicity of antibiotics like gentamicin, infectious agents like parasites, the oxidative stress modeled by ischemia-reperfusion in studies or exposing in electromagnetic fields (19-30). Previously some antioxidants in onion juice and watermelon extract or other herbal medicines have been investigated as improvers of sperm parameters in such conditions (31, 32).

Testicular freezing prior to chemotherapy (with medicines like cisplatin, busulfan or cyclophosphamide) and radiotherapy is of the ways of preserving fertility in children with cancer (19, 21, 33, 34). The freezing has two methods of slow-freezing (cryopreservation) and rapid-freezing (vitrification) (34-36). In order to perform these technics, it seems better to use SSCs instead of spermatozoa; because in patients who has not reached the puberty age, we do not have any spermatozoon, and also SSCs are not differentiated enough to have acrosomal vesicles, so they have lower metabolic activity and hence they are lower at exposed to abnormalities. However, the freezing-thawing process could result in damage and quality reduction of the cells (34). 

Melatonin has been found and proven in microorganisms such as primitive bacteria and green algae as a serve antioxidant.In addition, melatonin is also found in pluricellular organisms such as fungi, plants, insects, nematodes, and vertebrates like mammals (37). This hormone is secreted by the pineal gland and testes (38). Other than melatonin, ghrelin is a 28-amino acid endocrine peptide which is principally produced by the stomach (25-27, 39). Indeed ghrelin is a gut-brain hormone increasing food intake, inducing hunger and appetite via hypothalamic circuits. As well, this hormone causes the release of growth hormone and also induces adiposity in animal studies. In animal studies, central and peripheral administration of ghrelin activates the mesolimbic dopamine system mediating behaviors (40). Some new evidence highlight its role in reproductive functions regulation. In other words, this protein is not merely limited to gut and brain and has been detected previously in the tubular and interstitial compartments of testes of several vertebrate species (39).

After the narrative introduction presented above, we attempt to review the protective effects of melatonin and ghrelin on male reproduction system and in particular on SSCs, as two rather newly-discovered hormones.

## Materials and methods

For the present narrative review, we used the scientific databases and meta-search engines including Google Scholar, PubMed, Scopus and Science Direct, and Web of Science core collection. Since the related articles were rare, the time period was not important to us. As key words and contents of search, at first we searched for all the articles of the first and corresponding authors related to male's reproduction as the self-referring which is necessary for review articles; second, searched for "melatonin AND spermatogonia" and "ghrelin AND sperm"; and finally searched for general information about spermatogenesis process in order to write the introduction. There were some duplications between the first and the second levels. Our literature review method was summery-comparison matrix, a method helping researchers to extract relevant information from the literature, categorize and visualize them (41). 

## Results

The founded literatures are summarized in table I. The rows are sorted by time from the oldest to the newest. The results are from 1999 to 2016. Investigation of ghrelin on male reproduction system is a more emerging idea. These findings are discussed as below. 

**Table I:** The literature review matrix

**Hormone **		**Author, date (reference) **	**Main findings **
**Melatonin**			
	Badr et al, 1999 (60)		Single-dose injection of melatonin to mice, before irradiation, could preserve the fertility.
	d'Istria et al, 2003 (71)		Melatonin shows anti-proliferative effect in frags.
	Ghasemi et al, 2010 (68)		Melatonin may have a protective effect on busulfan induced testicular damage.
	Hemadi et al, 2009,11 (73,74)		Melatonin after thawing the vitrified tissue can lead to better maintenance of spermatogenesis kinetics and it may serve as a promising method for graft preservation.
	Gholami et al, 2013 (34)		The sertoli cell modeling of seminiferous tubes induced by 40 mg/kg busulfan, shows that melatonin can induce spermatogenesis. Melatonin induce apoptosis in the damaged cells underwent freezing-thawing process. Hence it can be used for cell screening during ART* process.
	Yang et al, 2014 (70)		This bovine study shows that melatonin has anti-apoptotic effects through up-regulation of spermatogenesis-related genes like *cyclin D1 *and *cyclin E*
	Gholami et al, 2014,15 (38,69)		Melatonin improves efficacy of SSC transplantation.
	Saki et al, 2016 (72)		In spite of the advantages of melatonin, it cannot prevent release of lactate dehydrogenase.
	Deng et al, 2016 (61)		Administration of melatonin results in differentiation of SSCs to haploid germ cells in vitro, other than the advantages mentioned before.
**Ghrelin**			
	Tena-Sempere, 2005 (75)		Expression of ghrelin was observed in Leydig cells and also its receptor, the growth hormone secretagogue receptor (GHSR) type 1a was observed in both Leydig and Sertoli cells. Ghrelin dose-dependently inhibit testosterone secretion in vitro and modulate proliferation of leydig cells in vivo, as well as the expression of *encoding stem cell factor* and other testis-related genes.
	Olejniczak et al, 2009 (76)		The expression site of ghrelin in seminiferous tubes can indicate the role of its in local regulation of spermatogenesis.
	Garcia et al, 2015 (78)		Ghrelin can prevent the sequels of cisplatin.
	Whirledge et al, 2015 (77)		Ghrelin can prevent the sequels of cisplatin. The receptor of ghrelin, the very GHSR type 1ahas also an important role. Thus it seems that GHSR agonists could be used for prevention from cisplatin-induced testicular damage.
	Taati et al, 2016 (25)		Ghrelin can improve sperm quality in the testes underwent ischemia-reperfusion injury.

The findings are currently based on animal studies

* ART: assisted reproductive technology

## Discussion

**Role of melatonin**

There are some ways introduced to maintain quality of the germ cells undergoing freezing-thawing process and also the quality of sperms and other germ cells; for instance, antioxidants such as Na_2_SeO_3_, quersetine or other flavonoid compounds, magnesium sulfate or antioxidants in medical plants like ginger and onion, carrot seed extract (56) or water melon seed, chemical drugs like tramadol and adding vitamins C and/or D in vitro (32, 42-58). Using melatonin is one of these methods.The quality of germ cells and sperms could be assayed by proteomic technics (59). 

The history of using melatonin dates back to the year 1999 when Badr, Habit and Harraz showed that single-dose injection of melatonin to mice, before irradiation, could preserve the fertility (60). From then on, there have been a few types of research in this application of its. The most recent study, shows that administration of melatonin results in differentiation of SSCs to haploid germ cells in vitro, other than the advantages mentioned before (61). Gholami and coworkers had shown this advantage through the Sertoli cell modeling of seminiferous tubes induced by 40 mg/kg busulfan (62). 

Melatonin is secreted by the pineal gland and testes (38). The fascinating feature of this hormone is that it makes the normal cells proliferate and makes the cancerous cells undergo apoptosis (62). Gholami* et al* showed that melatonin in mice induces apoptosis in the damaged cells underwent a freezing-thawing process (62). Although at the first, glans apoptosis has a negative connotation, hereby the cells are screened. This induction was through up-regulation of Fas and down-regulation of P53. Apoptosis is the most famous type of programmed cell death which is often physiologic and necessary like during implantation process of an embryo and sometimes is pathologic like secondary to a spinal cord trauma (63-67). Another example of physiologic apoptosis is maintaining the balance between the number of SSCs and gametes (68). Melatonin also improves the efficacy of SSC transplantation (38). Histopathologic studies show that melatonin reduces the oxidative injury to seminiferous tubes secondary to transplantation process in sheep (69). 

However, there are some controversies. For example, a bovine study shows that melatonin has anti-apoptotic effects through up-regulation of spermatogenesis-related genes like *cyclin D1 *and *cyclin E*, while another study shows anti-proliferative effect of melatonin in frogs (70, 71). Saki and the coworkers believe that in spite of the advantages of melatonin, it cannot prevent the release of lactate dehydrogenase (72). Moreover, Ghasemi *et al *believed that melatonin could have a protective effect on busulfan-induced testicular damage (68). Hemadi and the coworkers believe that use of melatonin after thawing the vitrified tissue can lead to better maintenance of spermatogenesis kinetics and it may serve as a promising method for graft preservation (73, 74). 

**Role of ghrelin**

In 2005, expression of ghrelin was observed in Leydig cells and also its receptor, the growth hormone secretagogue receptor (GHSR) type 1a was observed in both Leydig and Sertoli cells (75). In 2009 it has been shown that the expression site of ghrelin in seminiferous tubes can indicate the role of its in local regulation of spermatogenesis (76). This hormone has been proven to dose-dependently inhibit testosterone secretion in vitro, and modulate proliferation of Leydig cells in vivo, as well as the expression of encoding stem cell factor and other testis-related genes (75).

Cisplatin is an anti-cancer medicine which induces apoptosis both in cancerous and normal cells (20, 21). The apoptotic activity of cisplatin is due to inhibition of P53-dependent DNA repair. Among the sequels of cisplatin, damage to the germ cells can be pointed out; for example, damage to SSCs. It has been observed that ghrelin can prevent this sequel (77, 78). The receptor of ghrelin, the very GHSR type 1ahas also an important role. Thus it seems that GHSR agonists could be used for prevention from cisplatin-induced testicular damage (77).

In addition to cisplatin-induced damage, the studies done by Taati and the coworkers during recent years, show the protective effects of ghrelin from ischemia-reperfusion damage. They found that malondialdehyde values were significantly lowered in the treated group, and ghrelin significantly enhanced sperm movement, motility, and concentration. This protective effect is due to anti-apoptotic and anti-inflammatory effects of ghrelin (25-27). It seems that the similar feature of melatonin and ghrelin is their inhibitory role in mammalian reproduction.

## Conclusion

Infertility is a problem for a lot of couples. The male-based ones, either in real clinics or in research modeling, could be due to chemotherapy, radiotherapy, electromagnetic fields, ischemia-reperfusion and high oxidative stress, microbial and parasitic agents, or toxicity of antibiotics. This review indicates that in vitro or in vivo administration of melatonin and ghrelin as two newly discovered hormones, could be effective to preserve fertilization in such cases, and also they can be used in assisted reproductive technologies to improve the quality of the sperms. Future original studies should be propelled toward human studies of course with observing the ethics. 

## Conflict of interest

Hereby we declare that there is no conflict of interest.
